# The metabolic effects of mirabegron are mediated primarily by β_3_‐adrenoceptors

**DOI:** 10.1002/prp2.643

**Published:** 2020-08-19

**Authors:** Nodi Dehvari, Masaaki Sato, Muhammad Hamza Bokhari, Anastasia Kalinovich, Seungmin Ham, Jie Gao, Huong T. M. Nguyen, Lynda Whiting, Saori Mukaida, Jon Merlin, Ling Yeong Chia, Denise Wootten, Roger J. Summers, Bronwyn A. Evans, Tore Bengtsson, Dana S. Hutchinson

**Affiliations:** ^1^ Department of Molecular Biosciences The Wenner‐Gren Institute Stockholm University Stockholm Sweden; ^2^ Drug Discovery Biology Monash Institute of Pharmaceutical Sciences Monash University Parkville Vic. Australia

**Keywords:** adipocyte, glucose, mirabegron, UCP1, β_3_‐adrenoceptor

## Abstract

The β_3_‐adrenoceptor agonist mirabegron is approved for use for overactive bladder and has been purported to be useful in the treatment of obesity‐related metabolic diseases in humans, including those involving disturbances of glucose homeostasis. We investigated the effect of mirabegron on glucose homeostasis with in vitro and in vivo models, focusing on its selectivity at β‐adrenoceptors, ability to cause browning of white adipocytes, and the role of UCP1 in glucose homeostasis. In mouse brown, white, and brite adipocytes, mirabegron‐mediated effects were examined on cyclic AMP, UCP1 mRNA, [^3^H]‐2‐deoxyglucose uptake, cellular glycolysis, and O_2_ consumption. Mirabegron increased cyclic AMP levels, UCP1 mRNA content, glucose uptake, and cellular glycolysis in brown adipocytes, and these effects were either absent or reduced in white adipocytes. In brite adipocytes, mirabegron increased cyclic AMP levels and UCP1 mRNA content resulting in increased UCP1‐mediated oxygen consumption, glucose uptake, and cellular glycolysis. The metabolic effects of mirabegron in both brown and brite adipocytes were primarily due to actions at β_3_‐adrenoceptors as they were largely absent in adipocytes derived from β_3_‐adrenoceptor knockout mice. In vivo, mirabegron increased whole body oxygen consumption, glucose uptake into brown and inguinal white adipose tissue, and improved glucose tolerance, all effects that required the presence of the β_3_‐adrenoceptor. Furthermore, in UCP1 knockout mice, the effects of mirabegron on glucose tolerance were attenuated. Thus, mirabegron had effects on cellular metabolism in adipocytes that improved glucose handling in vivo, and were primarily due to actions at the β_3_‐adrenoceptor.

## 1 INTRODUCTION

The β_3_‐adrenoceptor was for many years a target in pharmaceutical drug programs for the development of antiobesity or antidiabetic drugs due to its high expression in rodent adipocytes, where its activation results in increased lipolysis, thermogenesis, and weight loss.^1^, ^2^ Most screening was done in rodent models of obesity leading to the development of what turned out to be rodent‐selective β_3_‐adrenoceptor agonists, such as CL316243.^3^ Although these compounds were active in in vitro cell‐based assays expressing high levels of human β_3_‐adrenoceptor, they had little effect at physiological levels of expression^4^, ^5^ leading to failure of the compounds in phase 2 clinical trials for the treatment of obesity or diabetes. The major factors involved were lack of efficacy of β_3_‐adrenoceptor agonists at the human receptor and poor bioavailability.^6^ However, β_3_‐adrenoceptors have re‐emerged as a target for metabolic disease in the past 10 years since the identification of functional brown adipose tissue (BAT) in adult humans ^7^ and the discovery of brite (brown within white) adipocytes.^8^ Brite adipocytes (or beige adipocytes) are brown‐like adipocytes located predominately in white adipose tissue (WAT) depots, and the process of browning has been advocated as a means to treat obesity and metabolic disease.^8^ Stimulation of β_3_‐adrenoceptors in rodent brite adipocytes causes increased oxygen consumption by activation of UCP1 and increased glucose uptake,^9^, ^10^ thus identifying a novel mechanism for the development of antiobesity and antidiabetic therapeutics.

Recently, a human active β_3_‐adrenoceptor agonist, mirabegron (Astellas Pharma Inc), was approved for the treatment of overactive bladder syndrome, but also has potential to be used for obesity/metabolic disease.^11^ Although there is limited information on the metabolic effects of mirabegron, one recent study showed that chronic treatment of mice fed a high‐fat diet lowered body weight, fat mass, and improved glucose tolerance.^12^ In lean humans, acute mirabegron administration positively affected BAT activity and resting metabolic rate,^13^, ^14^ whereas in obese humans, chronic mirabegron treatment led to improved glucose tolerance and insulin sensitivity.^15^ However, while mirabegron is recognized as a β_3_‐adrenoceptor agonist, its selectivity has been questioned, and it does have off‐target actions.^11^ These include actions at α_1_‐ and β_1_‐adrenoceptors, muscarinic receptors, noradrenaline and dopamine transporters, organic cation transporters, p‐glycoprotein, and 2 cytochrome P450 enzymes.

This study therefore investigates the effects of mirabegron in brown, white, and brite adipocytes in vitro, and its effects on glucose utilization and thermogenesis in vivo. We have utilized β_3_‐ and β_1/2_‐adrenoceptor knockout mice to assess selectivity. Additionally, we have performed glucose tolerance tests in UCP1 knockout mice to address the role of this protein in mediating the acute effects of mirabegron on blood glucose levels in vivo.

## 2 MATERIALS AND METHODS

### 2.1 Materials

All cell culture consumables were from Thermo Fisher Scientific (Scoresby, Victoria, Australia). Human insulin (Actrapid) was obtained from Novo Nordisk. Mirabegron was obtained from Cayman Chemicals. CGP20712A, fatty‐free BSA, forskolin, ICI118551, (–)‐isoprenaline, and SR59230A were obtained from Sigma‐Aldrich. Rosiglitazone was obtained from Tocris Bioscience. All other reagents were of analytical quality.

### 2.2 Animals

All experiments complied with the Australian code for the care and use of animals for scientific purposes (National Health and Medical Council of Australia, 8th edition), or under European Union legislation, and experiments were reviewed and approved by either the Monash University Animal Ethics Committee (Australia; ethics numbers MIPS.2015.14, MIPS.2015.25, MIPS.2013.37) or the North Stockholm Animal Ethics Committee (Sweden; ethics number N155/15). All mice were allowed free access to food (in Australia, Ridley Mice breeder cube, 20% protein, 8.5% fat, and 71.5% carbohydrate; in Sweden, Altromin Maintenance diet for mice, 24% protein, 15% fat, and 61% carbohydrate) and water.

FVB/N mice were the wild‐type control for the β_3_‐adrenoceptor knockout^16^ mice, DBAxC57 mice were the wild‐type control for the β_1/2_‐adrenoceptor knockout^17^ mice, and Bl6/129sv mice were the wild‐type control for the UCP1 knockout mice. A summary of the number of mice used for each experiment is given in Table 1.

**Table 1:** Number of animals used by strain and experiment

	FVB/N	β_3_‐adrenoceptor knockout	DBAxC57	β_1/2_‐adrenoceptor knockout	C57Bl6/N	Bl6/129Sv	UCP1 knockout

Adipocyte cultures^a^: Glucose uptake	57	30	23	27			
Adipocyte cultures^a^: qPCR	20	20					
Adipocyte cultures^a^: Seahorse (OCR/ECAR)	12	7					
Adipocyte cultures^a^: Cyclic AMP	18	10					
In vivo glucose uptake	9	12					
In vivo oxygen consumption	21	13					
Glucose tolerance test	15	10	8	8		10	10
Islet isolation					6		
Total number of mice used per strain	152	102	31	35	6	10	10

^a^ Brown, brite, and white adipocytes were obtained from the same mice.

### 2.3 Adipocyte cell culture

Mice (FVB/N, β_3_‐adrenoceptor knockout, DBAxC57, or β_1/2_‐adrenoceptor knockout mice; 3 to 4 weeks old, either sex) were killed by CO_2_ inhalation followed by cervical dislocation, and BAT was isolated from the interscapular, cervical, and axillary depots, while WAT was isolated from the subcutaneous inguinal depot. Cells were prepared as described previously.^18^ The culture medium consisted of Dulbecco's Modified Eagle's Medium (DMEM) containing 25‐mM glucose, 10% (vol/vol) newborn calf serum, 2.4‐nM insulin, 25 µg/mL sodium ascorbate, 10‐mM HEPES, 4‐mM L‐glutamine, 50 U/mL penicillin, and 50 µg/mL streptomycin. For the generation of brite adipocytes from white adipocyte cells, the medium was supplemented with 1‐µM rosiglitazone from day 1 to day 7‐8. Adipocytes were grown at 37°C in 8% CO_2_. Experiments were conducted on day 7‐8.

### 2.4 CHO cell culture

Chinese Hamster ovary (CHO) cells stably expressing either the human ^19^ or mouse ^20^ β_3_‐adrenoceptor, the human β_1_‐adrenoceptor, or the human β_2_‐adrenoceptor were grown in 50:50 DMEM/Ham's F12 medium supplemented with 5% (vol/vol) fetal bovine serum (FBS) and 2‐mM L‐glutamine at 37°C in 5% CO_2_. After plating, cells were maintained in growth medium overnight before being serum starved for ~16 h prior to experimentation. Cells were routinely tested for mycoplasma.

### 2.5 L6 cell culture

L6 cells over expressing GLUT4 with a myc epitope (kindly provided by Amira Klip, Hospital for Sick Children, Toronto, Canada) were grown in DMEM supplemented with 4‐mM L‐glutamine, 10% (vol/vol) FBS, 100 U/mL penicillin, 100 mg/mL streptomycin, and 10‐µM HEPES at 37°C in 5% CO_2_. Cells were grown as myoblasts by ensuring that cells were kept at less than 70% confluency. Upon confluency (90%), differentiation was induced by lowering the FBS concentration to 2% (vol/vol) for 7 days, with media changed every 2 days.

### 2.6 Measurement of cyclic AMP levels

Adipocytes were grown in 96‐well plates for 7 days before being serum starved in DMEM/Hams F12 media containing 0.5% (w/vol) BSA, 2.4‐nM insulin, 25 µg/mL sodium ascorbate, 10‐mM HEPES, 4‐mM L‐glutamine, 50 U/mL penicillin, and 50 µg/mL streptomycin, with 1‐µM rosiglitazone for brite adipocytes only. CHO and L6 cells were grown in 96‐well plates. cAMP assays were performed as described previously ^10^ and cAMP content was measured (αScreen kits; PerkinElmer Life Sciences) according to the manufacturer's instructions. Forskolin (100 µM; this concentration produced ~16‐fold increase in cAMP levels) was used as positive control in all cAMP assays to ensure an intact Gs‐cAMP pathway (data not shown).

### 2.7 Reverse transcription‐qPCR

Adipocytes were grown in 6‐well plates for 7 days and were serum starved as described above. In the morning, media were replaced by DMEM containing 25‐mM glucose, 0.5% (w/vol) BSA, and 0.125‐mM sodium ascorbate for 30 min, before mirabegron (1 µM) was added for 24 h. The cells were then washed in warmed PBS, and plates were rapidly frozen at −80**°**C until use. Total RNA was extracted using Tri Reagent (Sigma‐Aldrich) as per the manufacturer's instructions. The yield and quality of RNA were assessed by measuring absorbance at 260 and 280 nm (Nanodrop ND‐1000 Spectrophotometer; NanoDrop Technologies LLC). All RNA samples were stored at −80**°**C. For preparation of cDNA, 0.5 µg of RNA was reverse‐transcribed using iScript Reverse Transcription Supermix for RT‐qPCR (Bio‐Rad) according to the manufacturer's instructions. Briefly, the reactions consisted of 2 µL of 5 × iScript reverse transcription supermix, 3‐µL DNase/RNase free water, and 0.5 µg of RNA, in a final volume of 10 µL in 200‐µL Eppendorf PCR tubes. Reactions were performed on an Applied Biosystems 2720 Thermal Cycler (Applied Biosystems) as follows: 25°C for 5 min, 42°C for 30 min, 85°C for 5 min, and then cooled to 4°C. The cDNA was diluted with 190‐µL DNase/RNase free water to obtain the equivalent of 2.5 ng/µL of starting RNA, and stored at −20°C.

qPCR was performed in duplicate using TaqMan Gene Expression assays (Life Technologies) for Ucp1 (Mm01244861_m1) and Actb (β‐actin; Mm01205647_g1). Each reaction consisted of 4‐µL cDNA, 0.5‐µL TaqMan Gene Expression Assay, 0.5‐µL DNAse/RNase free water, and 5‐µL TaqMan Fast Advanced Master Mix dispensed in Eppendorf twin.tec PCR plates. qPCR reactions were carried out using a CFX Connect^TM^ Real‐Time PCR Detection System (Bio‐Rad). After initial heating at 50°C for 2 min and denaturation at 95°C for 10 min, fluorescence was detected over 40 cycles (95°C for 15 s and 60°C for 1 min). *C*_q_ values were automatically calculated by the Bio‐Rad analysis module. Data are expressed as expression of the gene of interest relative to Actb, calculated as (2^‐ΔCq^) × 1000. All statistics for gene expression were performed on Δ*C*_q_ values, as these data are normally distributed. MIQE guidelines were followed.

### 2.8 Measurement of oxygen consumption and extracellular acidification rates

Oxygen consumption rates (OCRs) and extracellular acidification rates (ECARs) were measured using the Seahorse xF96 (Seahorse Bioscience). Adipocytes were grown in Seahorse cell culture plates as above. On day 7, adipocytes were washed twice in XF assay medium (Seahorse Bioscience) supplemented with 25‐mM glucose, 0.5‐mM sodium pyruvate, 2‐mM L‐glutamine and 1% (w/vol) fatty acid‐free BSA, and 180‐µL added/well. OCR and ECAR were measured ^21^ with some modifications.^10^ Six baseline rate measurements were made using a 2 min mix, 5 min measure cycle. Agonists (20 µL) were injected pneumatically by the machine into each well, mixed, and 10 measurements were made using the 2 min mix, 5 min measure cycle. OCR and ECAR rates immediately prior to compound injection were used as the basal rates and defined as 100%.

### 2.9 [^3^H]‐2‐deoxyglucose uptake

Glucose uptake in adipocytes was performed as previously described.^10^, ^22^, ^23^ On day 6, cells were serum starved overnight in DMEM/Hams F12 media containing 0.5% (w/vol) BSA, 2.4‐nM insulin, 25 µg/mL sodium ascorbate, 10‐mM HEPES, 4‐mM L‐glutamine, 50 U/mL penicillin, and 50 µg/mL streptomycin, with 1‐µM rosiglitazone for brite adipocytes only. The following morning, medium was replaced to DMEM containing 0.5% (w/vol) BSA and 0.125‐mM sodium ascorbate for at least 30 min, before drugs were added for 2 h (where antagonists were used, they were added 30 min before the addition of agonists). Cells were then washed twice in prewarmed 37°C PBS and glucose‐free DMEM containing 0.5% (w/vol) BSA added. Drugs and trace amounts of 2‐deoxyglucose (50 nM; Perkin Elmer; specific activity 9.5‐12 Ci/mmol) were added for 10 min. Reactions were terminated by washing twice in ice‐cold PBS, cells lysed (500 μL of 0.2 M NaOH, 1 h at 55°C) and the incorporated radioactivity determined by liquid scintillation counting (Tri‐Carb 2900TR; PerkinElmer). Results are expressed as % of basal glucose uptake in each plate.

### 2.10 Glucose stimulation of mouse isolated islets

Mice (8‐week‐old male C57Bl6/N) were killed by cervical dislocation under anesthetic (isoflurane 2%‐5%) and collagenase solution (Collagenase P, Roche) at 1.3 IU/mL infused into the common bile duct after occlusion of the distal end just proximal to the duodenum. Pancreas was excised, and incubated in a water bath at 37°C for 10 min. At the end of digestion, the tissue was shaken for 1 minute and washed with HBSS (in mM: NaCl 138; KCl 5.3; KH_2_PO_4_ 0.4; Na_2_HPO_4_.7H_2_O 0.3; MgCl_2_.6H_2_O 0.5; MgSO_4_.7H_2_O 0.4; CaCl_2_ 1.3; glucose 5.6; pH 7.4) containing 20‐mM HEPES. Tube contents were filtered through a 500μm plastic mesh to discard undigested tissue and washed in RPMI 1640 (Life Technologies). RPMI was removed after centrifugation (200 ***g***, 2 min) and the pellet was resuspended in Histopaque (Sigma). RPMI media were overlaid and tubes were centrifuged (800 ***g***, 15 min). Islets were collected from the RPMI‐Histopaque interface and transferred into Connaught Medical Research Laboratories Medium (CMRL) medium (Life Technologies) containing 10% (vol/vol) FBS and 1% (vol/vol) penicillin/streptomycin. Islets were handpicked into fresh CMRL before being incubated for 48 h in a humidified incubator (5% CO_2_, 37°C). Islets were plated at 3 islets/well and insulin secretion was measured by static incubation using Earle's balanced salt solution (EBSS; in mM: NaCl, 117; NaHCO_3_, 26; KCl, 5.3; NaH_2_PO_4_, 1; MgSO_4_, 0.8; CaCl_2_, 1.8), containing 0.1% (w/vol) BSA. The incubation experiments were started with a 2 h preincubation of islets in EBSS with 2.8‐mM glucose. Islets were then incubated for 2 h in EBSS supplemented with 16.7‐mM glucose and different concentrations of mirabegron (100 pM–1 µM) or forskolin (1 µM). The medium was collected, and insulin was measured (HTRF insulin kit; Cisbio). Experiments were repeated three times in triplicate, with two mice being used for each individual *n* number.

### 2.11 Glucose tolerance tests

Mice (12‐week‐old male FVB/N, β_3_‐adrenoceptor knockout, DBAxC57, or β_1/2_‐adrenoceptor knockout mice; and 6 to 8‐week‐old male Blb/129Sv and UCP1 knockout mice) were starved for 5‐7 h before either mirabegron (1 mg/kg i.p., 20% Dimethyl sulfoxide (DMSO) in saline) or vehicle (20% DMSO in saline i.p.) was administered. Thirty minutes following mirabegron or vehicle treatment, glucose levels were measured by sampling ~5 µL of tail vein blood, before animals were challenged with glucose (2 g/kg i.p. in saline), and serial blood glucose levels measured. At the end of the experiment, animals were killed by CO_2_ inhalation and cervical dislocation.

### 2.12 Whole body oxygen consumption

Oxygen consumption was measured to determine non‐shivering thermogenic capacity following mirabegron injection using a Somedic INCA apparatus, principally as described previously.^24^ FVB/N and β_3_‐adrenoceptor knockout mice (12‐week‐old male mice) were anesthetized (85 mg/kg i.p. pentobarbital sodium) and placed in the chambers set to 30°C for ~25 min to estimate the basal metabolic rate. Thereafter, the mice were removed from the chamber briefly, injected with mirabegron (1 mg/kg i.p., 1.2% ethanol in saline) or vehicle (1.2% ethanol in saline), and placed back in the chambers for further measurements. At the completion of the experiment, mice were killed by CO_2_ inhalation and cervical dislocation.

### 2.13 In vivo glucose uptake

Mice (12‐week‐old male FVB/N and β_3_‐adrenoceptor knockout mice) were fasted for 5 hours before anesthesia (85 mg/kg i.p. pentobarbital sodium), then (~10 minutes) injected with mirabegron (1 mg/kg i.p., 1.2% ethanol in saline) or vehicle (1.2% ethanol in saline) and 130 μCi/kg of ^3^H‐2‐deoxyglucose (Perkin Elmer; 8 Ci/mmol). Animals were killed 1 hour later by CO_2_ inhalation and cervical dislocation. Tissues were dissected and lysed in 0.5 M NaOH at 60°C for at least 2 hours, and radioactivity was measured by liquid scintillation counting.

### 2.14 Statistical Analysis

All data are expressed as mean ± SEM of n. For concentration‐response data, curves were analyzed using nonlinear curve fitting (GraphPad Prism 7.02) to obtain pEC_50_ values. Statistical significance was determined by Student's t test, multiple comparisons one‐way ANOVA, or multiple comparisons Tukey's or Kruskal‐Wallis test (for nonparametric analyses), or a two‐way ANOVA with a Bonferroni post hoc test as indicated in results. *P* < .05 was considered significant.

## 3 RESULTS

### 3.1 Selectivity of mirabegron in CHO‐K1 cells expressing β‐adrenoceptor subtypes

**Figure 1:**
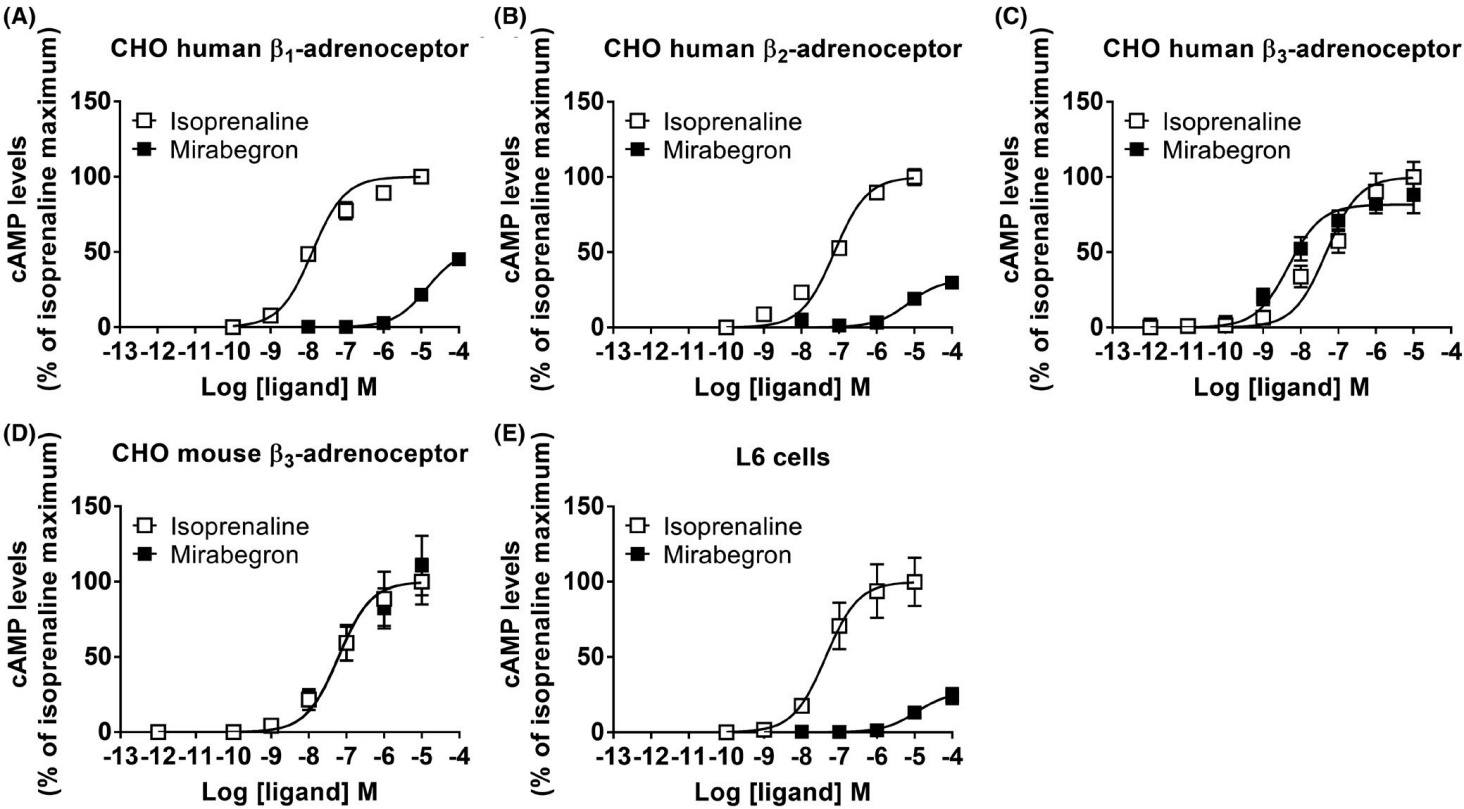
cAMP responses to mirabegron in recombinant cells. Cyclic AMP accumulation in response to mirabegron or isoprenaline in CHO‐K1 cells expressing the (A) human β_1_‐adrenoceptor (n = 5), (B) human β_2_‐adrenoceptor (n = 5), (C) human β_3_‐adrenoceptor (n = 4), (D) mouse β_3_‐adrenoceptor (n = 6), or in (E) rat L6 skeletal muscle cells that express the rat β_2_‐adrenoceptor (n = 5). Data are mean ± SEM of 4‐6 independent experiments performed in duplicate. All results are expressed as the maximal response to isoprenaline (defined as 100%)

To determine the selectivity of mirabegron at cloned human and mouse β‐adrenoceptor subtypes, its ability to generate cAMP was measured in CHO cells expressing the human β_1_‐, β_2_‐, or β_3_‐adrenoceptor, or the mouse β_3_‐adrenoceptor (Figure 1; Table 2). Mirabegron increased cAMP levels in all CHO‐K1 cells expressing β‐adrenoceptor subtypes, but was a weak partial agonist at human β_1_‐ and β_2_‐adrenoceptors compared with the prototypical β‐adrenoceptor agonist isoprenaline. It was ~2800 and ~1380 selective for the human β_3_‐ compared to β_1_‐ or β_2_‐adrenoceptors, consistent with previous results.^4^ It was a full agonist at the human and the mouse β_3_‐adrenoceptors. Thus, at cloned receptors, mirabegron is a potent and β_3_‐selective adrenoceptor agonist.

**Table 2:** Ability of mirabegron to increase cAMP levels in cells expressing different β‐adrenoceptor subtypes

Cell	Receptor	Ligand	Maximal effect^a^	pEC_50_	n
CHO‐K1	Human β_1_‐adrenoceptor	Isoprenaline	100.0 ± 4.5%	7.89 ± 0.08	5
		Mirabegron	51.7 ± 1.7%	4.85 ± 0.05	5
CHO‐K1	Human β_2_‐adrenoceptor	Isoprenaline	100.0 ± 5.4%	7.12 ± 0.08	5
		Mirabegron	32.0 ± 2.8%	5.16 ± 0.16	5
CHO‐K1	Human β_3_‐adrenoceptor	Isoprenaline	100.0 ± 10.1%	7.32 ± 0.14	4
		Mirabegron	81.7 ± 3.8%	8.32 ± 0.14	4
CHO‐K1	Mouse β_3_‐adrenoceptor	Isoprenaline	100.0 ± 15.2%	7.21 ± 0.18	6
		Mirabegron	93.8 ± 7.8%	7.25 ± 0.20	6
L6	Rat β_2_‐adrenoceptor	Isoprenaline	100.0 ± 16.1%	7.35 ± 0.21	5
		Mirabegron	26.9 ± 3.6%	4.95 ± 0.22	5

^a^ Defined as a percentage of the response to isoprenaline in each experiment.

### 3.2 Mirabegron increases β_3_‐adrenoceptor‐mediated glucose clearance in BAT and inguinal WAT

**Figure 2:**
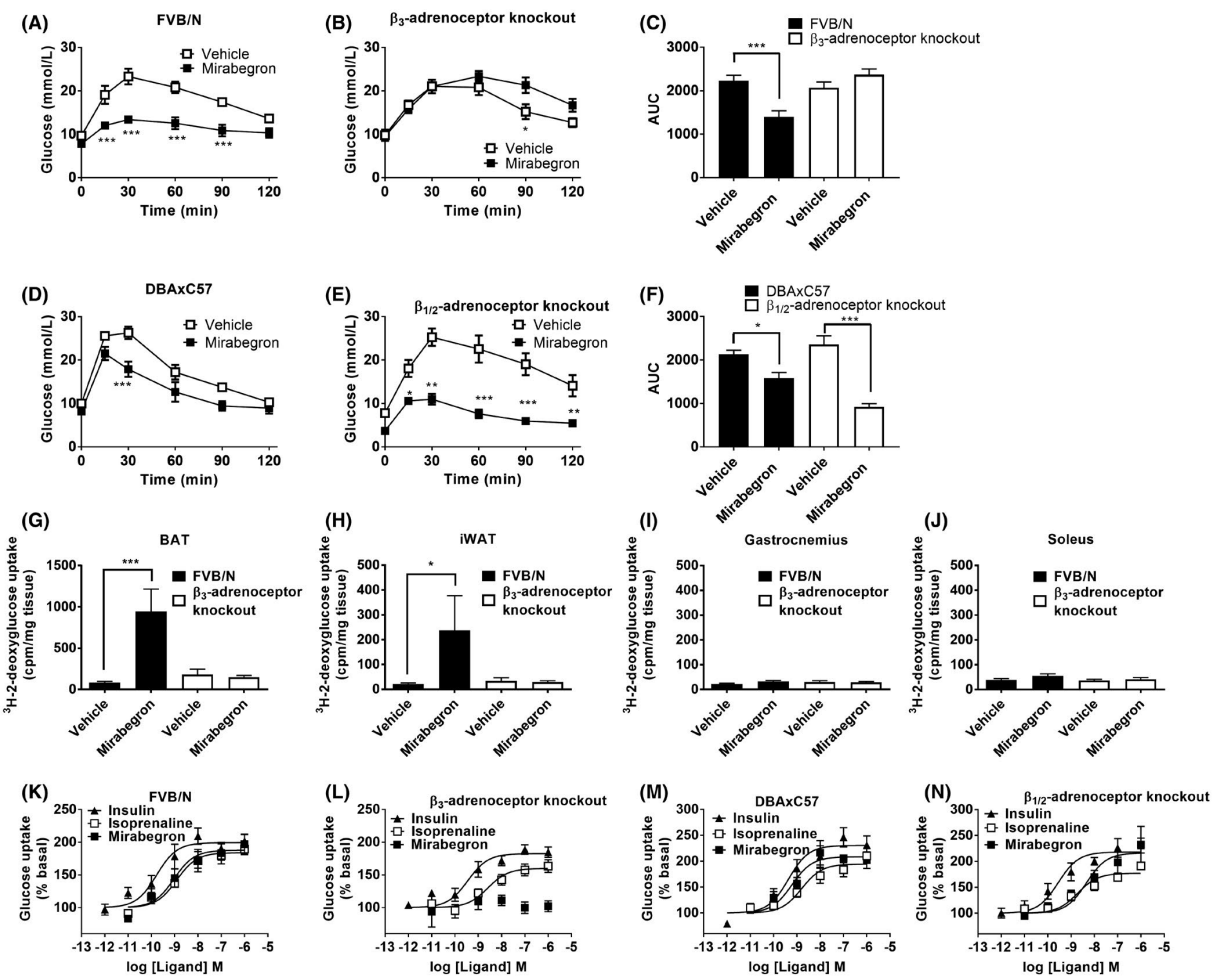
Mirabegron improves glucose tolerance, and increases brown adipocyte glucose uptake via activation of β_3_‐adrenoceptors. (A) In FVB/N mice, mirabegron (1 mg/kg i.p.; n = 9) administration 30 min prior to a glucose challenge (2 g/kg i.p.) significantly reduced blood glucose levels at 15, 30, 60, and 90 min compared with control‐treated (20% DMSO in saline, i.p.; n = 6) FVB/N mice. (B) In β_3_‐adrenoceptor knockout mice, mirabegron (1 mg/kg i.p.; n = 5) administration 30 min prior to a glucose challenge (2 g/kg i.p.) significantly increased blood glucose levels at 90 min compared with control‐treated (20% DMSO in saline, i.p.; n = 5) β_3_‐AR KO mice. Data analyzed using a two‐way ANOVA with a Bonferroni post hoc test (*P < .05, ***P < .001). (C) Area under the curve analysis of glucose levels from (A) and (B) shows that mirabegron (1 mg/kg i.p) significantly reduces glucose levels in FVB/N but not β_3_‐adrenoceptor knockout mice. Data analyzed using unpaired t‐test (***P < .001). (D) In DBAxC57 mice, mirabegron (1 mg/kg i.p.; n = 4) administration 30 min prior to a glucose challenge (2 g/kg i.p.) significantly reduced blood glucose levels at 30 min compared with control‐treated (20% DMSO in saline, i.p.; n = 4) DBAxC57 mice. (E) In β_1/2_‐adrenoceptor knockout mice, mirabegron (1 mg/kg i.p.; n = 4) administration 30 min prior to a glucose challenge (2 g/kg i.p.) significantly decreased blood glucose levels at 15, 30, 60, and 90 min compared with control‐treated (20% DMSO in saline, i.p.; n = 4) β_1/2_‐adrenoceptor mice. Data analyzed using a two‐way ANOVA with a Bonferroni post hoc test (*P < .05, ***P < .001). (F) Area under the curve analysis of glucose levels from (D) and (E) shows that mirabegron (1 mg/kg i.p) significantly reduces glucose levels in both DBAxC57 and β_1/2_‐adrenoceptor knockout mice. Data analyzed using unpaired t‐test (***P < .001, *P < .05). ^3^H‐2‐deoxyglucose uptake into (G) BAT, (H) iWAT, (I) gastrocnemius, or (J) soleus in FVB/N mice and β_3_‐adrenoceptor knockout mice treated with either mirabegron (1 mg/kg i.p.; n = 4 FVB/N mice, n = 7 β_3_‐adrenoceptor knockout mice) or vehicle (n = 5 FVB/N mice, n = 5 β_3_‐adrenoceptor knockout mice). Glucose uptake was only significantly increased in BAT and iWAT following mirabegron treatment in FVB/N mice. Data analyzed using a one‐way ANOVA with a Tukey post hoc test (*P < .05, ***P < .001). Effect of insulin, isoprenaline, or mirabegron on glucose uptake in brown adipocytes derived from (K) FVB/N mice (n = 7), (L) β_3_‐adrenoceptor knockout mice (n = 6), (M) DBAxC57 mice (n = 5‐6), or (N) β_1/2_‐adrenoceptor knockout mice (n = 6‐7)

To determine the effects of acute mirabegron administration on glucose tolerance and whether this was mediated through β_3_‐adrenoceptors, FVB/N, DBAxC57, β_1/2_‐adrenoceptor, or β_3_‐adrenoceptor knockout mice were fasted for 5‐7 hours and an i.p. GTT was performed. Both mirabegron‐treated FVB/N mice and DBAxC57 mice exhibited marked improvements in glucose clearance compared to the control‐treated group (Figure 2A,D). Similarly, β_1/2_‐adrenoceptor knockout mice retained the ability to increase glucose disposal following mirabegron administration suggesting that β_1_‐ and β_2_‐adrenoceptors are not required for this effect (Figure 2E). However, in β_3_‐adrenoceptor knockout animals, mirabegron‐dependent improvements in glucose tolerance were completely abolished (Figure 2B).

In vivo glucose uptake assays were performed to gauge the contribution of metabolically active tissues toward improvements in glucose clearance observed in mirabegron‐treated mice. Mirabegron treatment was associated with a robust increase in ^3^H‐2‐deoxyglucose uptake into BAT (Figure 2G) and inguinal WAT (Figure 2H) with no increase observed in soleus and gastrocnemius muscles (Figure 2I,J), suggesting adipose tissue is the primary site of glucose clearance following mirabegron treatment. However, in β_3_‐adrenoceptor knockout mice, mirabegron failed to induce glucose uptake into BAT or inguinal WAT and, as with FVB/N mice, there were no mirabegron‐dependent increases in glucose uptake in soleus and gastrocnemius muscles (Figures 2G‐J), confirming that β_3_‐adrenoceptors are essential for induction of mirabegron‐mediated glucose uptake. Mirabegron also failed to increase cAMP levels in L6 skeletal muscle cells, suggesting a lack of effect in skeletal muscle (Figure 1; Table 2).

**Figure 3:**
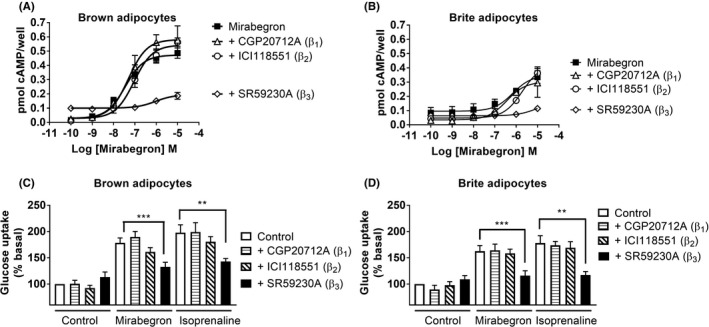
Selectivity of mirabegron using antagonists in brown and brite adipocytes. Effect of the β_1_‐adrenoceptor antagonist CGP20712A (300 nM), the β_2_‐adrenoceptor antagonist ICI118551 (300 nM), or the β_3_‐adrenoceptor antagonist SR59230A (300 nM) on cyclic AMP responses mediated by mirabegron in (A) brown or (B) brite adipocytes derived from FVB/N mice (n = 4). Effect of the β_1_‐adrenoceptor antagonist CGP20712A (300 nM), the β_2_‐adrenoceptor antagonist ICI118551 (300 nM), or the β_3_‐adrenoceptor antagonist SR59230A (300 nM) on glucose uptake mediated by mirabegron (100 nM) or isoprenaline (100 nM) in (D) brown (n = 6) or (E) brite (n = 6) adipocytes derived from FVB/N mice

To supplement in vivo findings, primary brown adipocytes were derived from FVB/N, DBAxC57, β_1/2_‐adrenoceptor, or β_3_‐adrenoceptor knockout mice and in vitro glucose uptake assays were performed. In cells from FVB/N, β_1/2_‐adrenoceptor knockout, and DBAxC57 mice, insulin, isoprenaline, and mirabegron all increased cellular glucose uptake in a concentration‐dependent manner (Table 3; Figure 2K,M,N). In primary cell cultures of brown adipocytes from β_3_‐adrenoceptor knockout mice, insulin and isoprenaline stimulation caused marked increases in glucose uptake, whereas mirabegron had no effect, highlighting the essential role of the β_3_‐adrenoceptor in the actions of mirabegron (Table 3; Figure 2L). Mirabegron also increased glucose uptake in brown adipocytes derived from FVB/N mice pretreated with a β_1_‐ or β_2_‐antagonist but failed to do so after treatment with a β_3_‐adrenoceptor antagonist (Figure 3A,B). These results strongly suggest that the effects of mirabegron on glucose uptake in brown adipocytes are mediated through the β_3_‐adrenoceptor. Taken together, the results suggest that acute administration of mirabegron markedly improves glucose tolerance by increasing glucose uptake into BAT and inguinal WAT following β_3_‐adrenoceptor activation.

**Table 3:** Ability of mirabegron, isoprenaline, and insulin to increase glucose uptake in brown, brite, and white adipocytes

Mouse strain	Adipocyte	Ligand	Maximal effect^a^	pEC_50_	n
FVB/N	Brown	Isoprenaline	184.1 ± 5.7%	8.92 ± 0.19	7
		Mirabegron	187.8 ± 8.3%	9.03 ± 0.26	7
		Insulin	199.4 ± 6.6%	9.78 ± 0.21	7
	White	Isoprenaline	NA	NA	6
		Mirabegron	NA	NA	8
		Insulin	173.7 ± 4.4%	10.29 ± 0.20	6
	Brite	Isoprenaline	165.2 ± 5.3%	8.98 ± 0.22	6
		Mirabegron	149.1 ± 4.9%	8.33 ± 0.25	9
		Insulin	182.0 ± 4.9%	9.42 ± 0.18	6
β_3_‐adrenoceptor knockout	Brown	Isoprenaline	159.9 ± 5.1%	8.59 ± 0.23	6
		Mirabegron	NA	NA	6
		Insulin	182.5 ± 5.1%	9.45 ± 0.19	6
	White	Isoprenaline	NA	NA	4
		Mirabegron	NA	NA	4
		Insulin	185.3 ± 9.3%	8.88 ± 0.28	4
	Brite	Isoprenaline	136.6 ± 3.9%	8.64 ± 0.29	4
		Mirabegron	NA	NA	5
		Insulin	203.2 ± 10.8%	9.90 ± 0.32	4
DBAxC57	Brown	Isoprenaline	194.6 ± 7.8%	8.79 ± 0.22	6
		Mirabegron	208.6 ± 8.6%	9.21 ± 0.23	6
		Insulin	230.6 ± 9.3%	9.36 ± 0.22	5
β_1/2_‐adrenoceptor knockout	Brown	Isoprenaline	177.3 ± 5.2%	8.72 ± 0.18	6
		Mirabegron	216.4 ± 13.5%	8.35 ± 0.30	7
		Insulin	218.2 ± 8.0%	9.55 ± 0.21	7

Note

NA, no response.

^a^ Basal glucose uptake defined as 100%.

### 3.3 Mirabegron administration causes β_3_‐adrenoceptor‐mediated thermogenic activation of BAT

**Figure 4:**
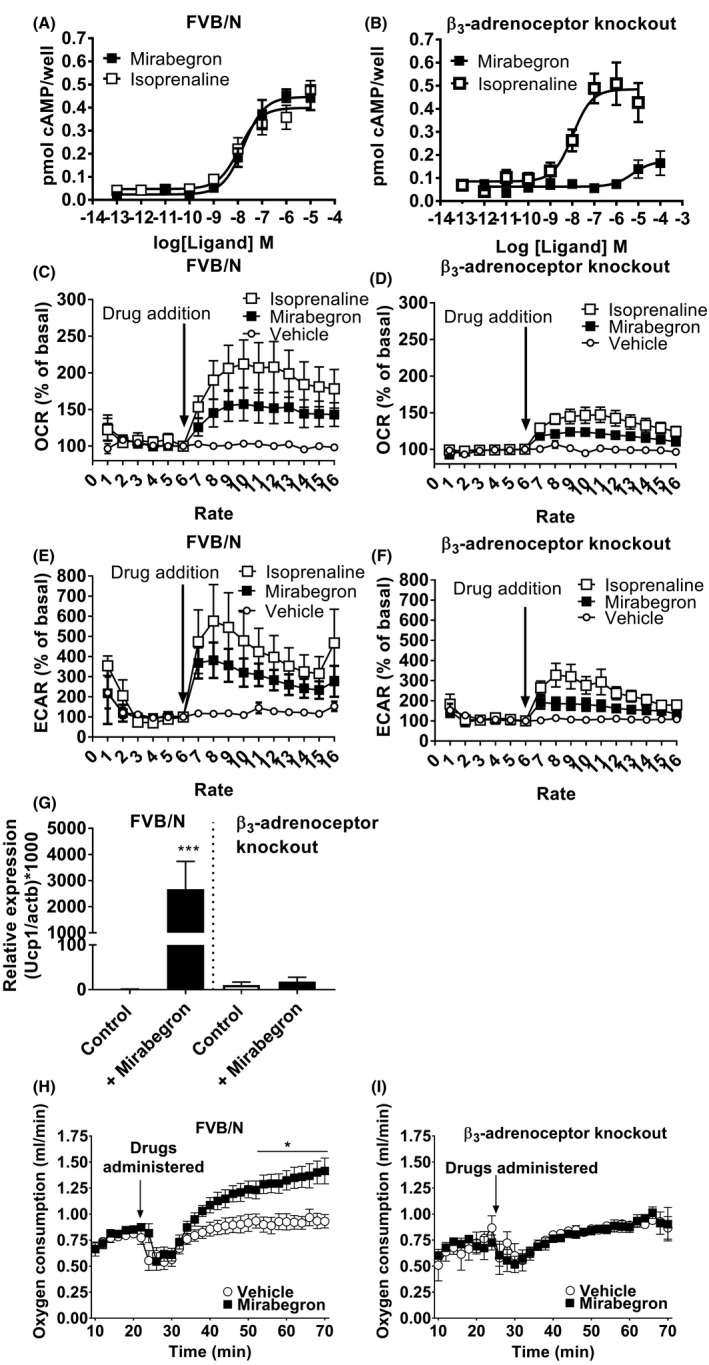
Mirabegron increases cAMP levels, OCR and ECAR rates, and UCP1 mRNA levels in brown adipocytes via activation of β_3_‐adrenoceptors. Cyclic AMP accumulation in response to isoprenaline or mirabegron in adipocytes derived from (A) FVB/N (n = 5) or (B) β_3_‐adrenoceptor knockout (n = 7) mice. (C, D) Effect of acute addition of isoprenaline (1 µM) or mirabegron (1 µM) on OCR in brown adipocytes derived from FVB/N (n = 6 isoprenaline; n = 12 mirabegron) or β_3_‐adrenoceptor knockout (n = 6 isoprenaline; n = 5 mirabegron) mice. (E, F) Effect of acute addition of isoprenaline (1 µM) or mirabegron (1 µM) on ECAR in brown adipocytes derived from FVB/N (n = 5 isoprenaline; n = 11 mirabegron) or β_3_‐adrenoceptor knockout (n = 6 isoprenaline; n = 6 mirabegron) mice. (G) Effect of mirabegron (1 µM, 24 h) on Ucp1 mRNA expression in brown adipocytes derived from FVB/N (n = 6) or β_3_‐adrenoceptor knockout (n = 5) mice. Data are mean ± SEM of 5‐6 independent experiments performed in duplicate. Data analyzed using Student's paired t‐test (***P < .001). (H) In FVB/N mice, mirabegron (1 mg/kg i.p.; n = 9) administration significantly increased O_2_ consumption rates in FVB/N mice as compared to vehicle‐treated mice (n = 12). (I) In β_3_‐adrenoceptor knockout mice, mirabegron (1 mg/kg i.p.; n = 7) administration had no effect on O_2_ consumption rates as compared to vehicle‐treated mice (n = 6). Data analyzed using a two‐way ANOVA with a Bonferroni post hoc test (*P < .05)

cAMP production is a major determinant of BAT thermogenic activity.^2^ To examine whether mirabegron increased cAMP levels in these cells, primary brown adipocytes were isolated from FVB/N or β_3_‐adrenoceptor knockout mice and treated with isoprenaline or mirabegron. In primary brown adipocytes derived from FVB/N mice, mirabegron and isoprenaline increased cAMP levels in a concentration‐dependent manner, whereas responses to mirabegron but not isoprenaline were attenuated in adipocytes derived from β_3_‐adrenoceptor knockout mice (Table 4; Figure 4A,B).

**Table 4:** Ability of mirabegron and isoprenaline to increase cAMP levels in brown, brite, and white adipocytes

Mouse strain	Adipocyte	Ligand	Maximal effect^a^	pEC_50_	n
FVB/N	Brown	Isoprenaline	0.40 ± 0.02	7.93 ± 0.17	5
		Mirabegron	0.45 ± 0.02	7.76 ± 0.17	5
	White	Isoprenaline	0.19 ± 0.03	6.96 ± 0.57	5
		Mirabegron	NA	NA	5
	Brite	Isoprenaline	0.37 ± 0.02	7.26 ± 0.19	5
		Mirabegron	0.33 ± 0.03	6.42 ± 0.26	5
β_3_‐adrenoceptor knockout	Brown	Isoprenaline	0.48 ± 0.03	7.98 ± 0.26	7
		Mirabegron	0.17 ± 0.01	5.27 ± 0.51	7
	White	Isoprenaline	0.15 ± 0.02	7.34 ± 0.75	6
		Mirabegron	0.11 ± 0.02	7.37 ± 1.00	6
	Brite	Isoprenaline	0.17 ± 0.01	7.72 ± 0.29	6
		Mirabegron	NA	NA	6

Note

NA, no response.

^a^ Expressed in pmol/well.

To determine whether increases in cAMP production following mirabegron in vitro translated into increased thermogenic activity, OCR was measured in primary brown adipocytes using plate‐based respirometry. Experiments were performed in the presence of 1% fatty acid‐free BSA to minimize UCP1‐independent effects due to exogenous fatty acids and to fatty acids liberated by lipolysis.^25^ Isoprenaline and mirabegron were both able to increase OCR in primary brown adipocytes derived from FVB/N mice (Figure 4C). In brown adipocytes derived from β_3_‐adrenoceptor knockout mice, the effects of isoprenaline were still evident most likely due to its effects on β_1_‐adrenoceptors that can compensate for the lack of β_3_‐adrenoceptors in these cells.^23^ Interestingly, mirabegron was still able to increase OCR in brown adipocytes derived from β_3_‐adrenoceptor knockout mice (Figure 4D), albeit this response was modest compared to that in brown adipocytes derived from FVB/N mice.

ECAR was measured in tandem using the Seahorse xF96 analyzer as a proxy for glycolytic activity in these cells where pyruvate is reduced to lactate. In adipocytes derived from FVB/N mice, isoprenaline and mirabegron treatment increased ECAR several fold in brown adipocytes (Figure 4E). Noticeably, isoprenaline and mirabegron still increased ECAR in brown adipocytes derived from β_3_‐adrenoceptor knockout mice, although the effect was much smaller than in brown adipocytes derived from FVB/N mice, suggesting a non‐β_3_‐adrenoceptor‐mediated effect of mirabegron and isoprenaline on glycolytic activity in these cells (Figure 4F).

Additionally, to investigate whether mirabegron could directly recruit BAT, UCP1 expression was measured in primary brown adipocytes from FVB/N mice where treatment significantly increased UCP1 mRNA. This effect was absent in brown adipocytes from β_3_‐adrenoceptor knockout mice (Figure 4G).

Finally, to determine if mirabegron‐mediated increases in cAMP and OCR were sufficient to increase whole body energy expenditure in vivo, indirect calorimetry was performed where FVB/N or β_3_‐adrenoceptor knockout mice were injected with vehicle or mirabegron (Figure 4H,I). Oxygen consumption was increased significantly by mirabegron in FVB/N but not in β_3_‐adrenoceptor knockout mice, showing that mirabegron recruits and activates BAT in a β_3_‐adrenoceptor‐dependent manner.

### 3.4 Mirabegron promotes browning and increases β_3_‐adrenoceptor‐dependent thermogenic activity in brite adipocytes

**Figure 5:**
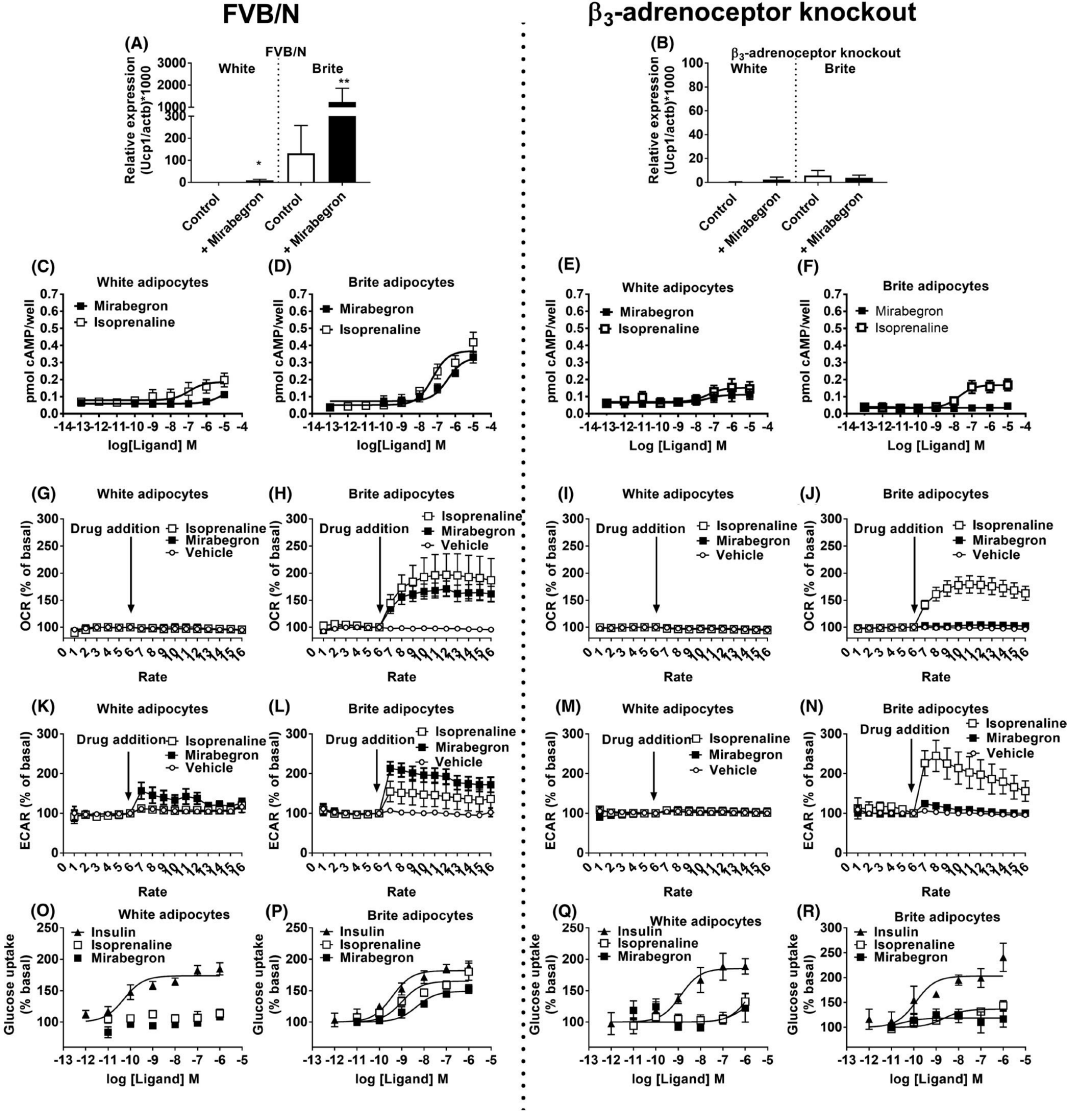
β_3_‐Adrenoceptors are required for the browning effect of mirabegron. (A, B) Effect of mirabegron (1 µM, 24 h) on Ucp1 mRNA expression in white and brite adipocytes derived from FVB/N (n = 5) or β_3_‐adrenoceptor knockout (n = 5) mice. Data are mean ± SEM of 5‐6 independent experiments performed in duplicate. Data analyzed using Student's paired t‐test (**P < .01, *P < .05). Cyclic AMP accumulation in response to isoprenaline or mirabegron in white and brite adipocytes derived from (C, D) FVB/N (n = 5) or (E, F) β_3_‐adrenoceptor knockout (n = 6) mice. Effect of acute addition of isoprenaline (1 µM) or mirabegron (1 µM) on OCRs (OCR) in white or brite adipocytes derived from (G, H) FVB/N (n = 5‐13) or (I, J) β_3_‐adrenoceptor knockout (n = 6‐7) mice. Effect of acute addition of isoprenaline (1 µM) or mirabegron (1 µM) on ECAR in white and brite adipocytes derived from (K, L) FVB/N (n = 5‐13) or (M, N) β_3_‐adrenoceptor knockout (n = 7) mice. Glucose uptake in response to isoprenaline, mirabegron, or insulin in white and brite adipocytes derived from (O,P) FVB/N (n = 6‐9) or (Q,R) β_3_‐adrenoceptor knockout (n = 4‐5) mice

Chronic mirabegron treatment is associated with browning of subcutaneous WAT in humans^15^ and inguinal WAT in rodents.^12^ To address the importance of the β_3_‐adrenoceptor for browning and thermogenic activation of these cells, white adipocytes derived from FVB/N or β_3_‐adrenoceptor knockout mice were treated with rosiglitazone for 7‐8 days to acquire a brite phenotype, that displayed upregulated levels of UCP1 expression relative to white adipocytes^9^, ^10^, ^26^ (Figure 5A). In brite adipocytes, mirabegron treatment markedly increased UCP1 expression. In the absence of the β_3_‐adrenoceptor, as observed in brown adipocytes, mirabegron had no effect on UCP1 expression in white or brite adipocytes (Figure 5B).

The effect of mirabegron and isoprenaline on cAMP, OCR, ECAR, and glucose uptake was measured in white and brite adipocytes derived from FVB/N or β_3_‐adrenoceptor knockout mice. Isoprenaline increased cAMP levels in a concentration‐dependent manner in white adipocytes from both FVB/N and β_3_‐adrenoceptor knockout mice, and had a larger effect in brite adipocytes^10^ (Figure 5C‐F). Mirabegron had little effect on cAMP levels in white adipocytes and only increased cAMP levels in brite adipocytes derived from FVB/N and not those from β_3_‐adrenoceptor knockout mice (Figure 5C‐F).

Both mirabegron and isoprenaline increased OCR and ECAR in brite adipocytes derived from FVB/N mice (Figure 5H,L), with the effects of mirabegron almost abolished in brite adipocytes from β_3_‐adrenoceptor knockout mice (Figure 5J,N). In white adipocytes derived from FVB/N or β_3_‐adrenoceptor knockout mice, increases in OCR and ECAR were largely absent (Figure 5G,K,I,M).

Treatment with insulin resulted in increased glucose uptake into white and brite adipocytes derived from both FVB/N and β_3_‐adrenoceptor knockout mice (Figure 5O‐R). Isoprenaline and mirabegron were only effective in increasing glucose uptake in brite adipocytes derived from FVB/N but not β_3_‐adrenoceptor knockout mice. These results were further validated using β‐adrenoceptor antagonists in FVB/N‐derived brite adipocytes (Figure 3B,D).

### 3.5 Mirabegron‐mediated improvements in glucose tolerance are UCP1‐dependent but insulin‐independent in vivo

**Figure 6:**
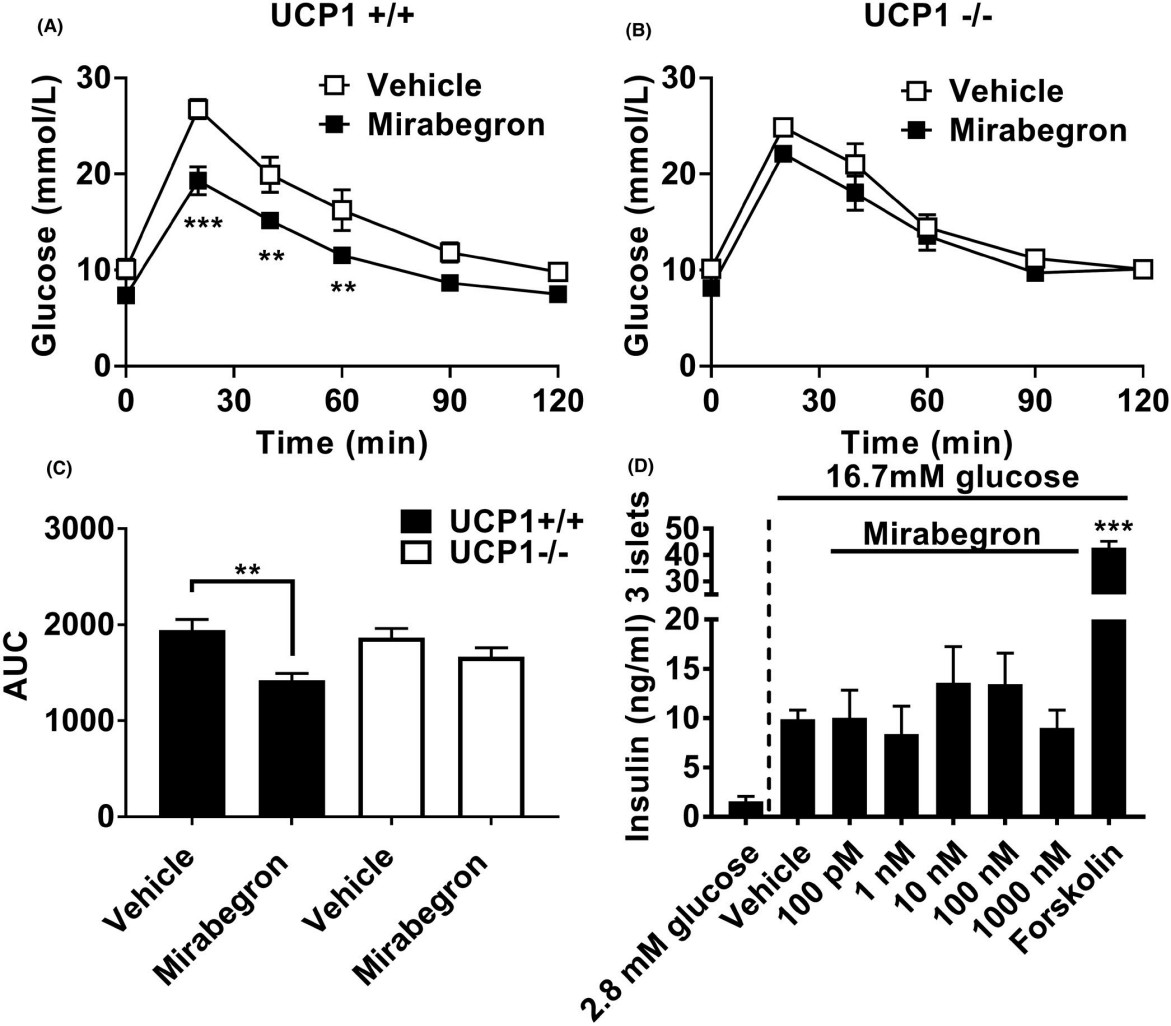
UCP1 is required for improved in vivo glucose tolerance in response to mirabegron. (A) In UCP1+/+ mice, mirabegron (1 mg/kg i.p.; n = 6) administration 30 min prior to a glucose challenge (2 g/kg i.p.) significantly reduced blood glucose levels at 20, 40, and 60 min compared with control‐treated (20% DMSO in saline, i.p.; n = 4) UCP1+/+ mice. (B) In UCP1–/– mice, mirabegron (1 mg/kg i.p.; n = 5) administration 30 min prior to a glucose challenge (2 g/kg i.p.) had no effect on blood glucose levels compared with control‐treated (20% DMSO in saline, i.p.; n = 5) UCP1–/– mice. Data analyzed using a two‐way ANOVA with a Bonferroni post hoc test (**P < .01). (C) Area under the curve analysis of glucose levels from panels A and B shows that mirabegron (1 mg/kg i.p) significantly reduces glucose levels in UCP1+/+ but not in UCP1−/− mice. Data analyzed using unpaired t‐test (**P < .001). (D) Mirabegron does not affect glucose‐stimulated insulin secretion in isolated mice islets. Each bar represents mean ± SEM from three incubations of 3 islets/well performed in three separate experiments. Forskolin (1 µM) increases glucose‐stimulated insulin secretion compared with untreated islets (***P < .001 compared with control) (one‐way ANOVA with Tukey multiple comparison test). In contrast, mirabegron at all concentrations did not affect stimulated insulin compared with untreated islets

Our results show that mirabegron activates thermogenesis and increases glucose uptake into brown and brite adipocytes, and improves glucose tolerance in vivo highlighting the feasibility of β_3_‐adrenoceptors as a target in metabolic disease. To address whether UCP1 is required for the observed improvements in glucose tolerance with mirabegron, UCP1 knockout and wild‐type controls were treated with mirabegron 30 minutes prior to glucose challenge. Mirabegron treatment produced significant improvements in glucose clearance in wild‐type mice but had no effect in UCP1 knockout animals (Figure 6A‐C).

Given previous findings that mirabegron has off‐target activity,^11^ we confirmed that the improved glucose tolerance was not due to a direct effect on insulin release from pancreatic islets. Incubation of islets in high glucose conditions (16.7 mM) increased insulin secretion compared to low glucose conditions (2.8 mM) (Figure 6D). Forskolin increased glucose‐stimulated insulin secretion in mouse islets compared to islets incubated with 16.7‐mM glucose alone (Figure 6D). Mirabegron did not have an additional effect on insulin secretion compared with insulin secreted from islets treated with 16.7‐mM glucose alone (Figure 6D). Since mirabegron does not directly affect insulin release from the pancreas, the acute effects of mirabegron on blood glucose clearance in vivo are therefore fully dependent on its effects on UCP1.

## 4 DISCUSSION

Although the main role for BAT is thermogenesis,^2^ it also represents an important glucose‐clearing organ displaying significant glucose uptake per gram of tissue, that in cold‐exposed obese mice contributes ~75% of whole body glucose uptake.^27^ In chow and high‐fat diet‐fed mice, BAT transplantation decreases body weight, increases glucose metabolism and insulin sensitivity, and increases glucose uptake into BAT and WAT.^28^ This makes BAT an attractive target for treating hyperglycemia associated with metabolic disease. We show here that mirabegron increases glucose uptake and glycolysis in mouse brown adipocytes in vitro and promotes glucose uptake into BAT in vivo. This results in rapid large improvements in glucose tolerance, likely attributable to increased glucose uptake into BAT, rather than skeletal muscle, another major site for glucose disposal. It has been suggested that β_3_‐adrenoceptor‐mediated glucose uptake into BAT occurs indirectly through an insulin‐dependent mechanism, whereby β_3_‐adrenoceptor‐mediated lipolysis leads to pancreatic insulin release, that then increases glucose uptake into BAT.^29^, ^30^ Our results do not support the involvement of insulin since mirabegron in vivo would then be expected to also increase glucose uptake into skeletal muscle, an effect that was not observed. Additionally, we observed no direct actions of mirabegron on glucose‐stimulated insulin secretion from isolated pancreatic islets.

While mirabegron was developed as a human β_3_‐adrenoceptor agonist, it has off‐target actions on heart rate that could be due to actions at cardiac β‐adrenoceptors,^11^ but may also be indirect due to release of noradrenaline.^31^ In one study of the metabolic effects of mirabegron in humans where large doses (four times the usual therapeutic dose) were given, unwanted increases in heart rate and systolic blood pressure were all too evident.^14^ Here, we examined the β‐adrenoceptor selectivity of mirabegron in CHO cells expressing human β‐adrenoceptor subtypes, in cultured mouse adipocytes and in vivo using mice with genetic deletion of either the β_3_‐adrenoceptor or combined β_1/2_‐adrenoceptor. All responses to mirabegron were lost or substantially depleted in the β_3_‐adrenoceptor knockout mice, but unaffected in the β_1/2_‐adrenoceptor knockout animals. The small but significant residual cAMP, OCR, and ECAR responses to mirabegron occurred only in brown adipocytes derived from β_3_‐adrenoceptor knockout mice. It has been shown previously that mirabegron acts as a partial agonist at rat β_1_‐adrenoceptors (intrinsic activity 0.6 relative to isoprenaline, pEC_50_ 6.21), despite having negligible direct activity at the human β_1_‐adrenoceptor, and at β_2_‐adrenoceptors from both species.^4^, ^32^ Since cultured mouse brown adipocytes express β_1_‐adrenoceptor mRNA,^10^ it is likely that the residual responses to mirabegron in β_3_‐adrenoceptor knockout mice are mediated by the β_1_‐adrenoceptor. Interestingly, a recent paper ^33^ suggested that the β_1_‐ and not the β_3_‐adrenoceptor is the primary adrenergic regulator of human brown adipocyte metabolism. Using a human immortalized brown adipocyte cell model, they showed that mirabegron had no effect on lipolysis or on the expression of several genes including UCP1.^33^ This correlated with little expression of β_3_‐adrenoceptors at the mRNA level, and supports our finding that mirabegron is a β_3_‐selective adrenoceptor agonist (Figure 1). In human brown adipocytes, the effects of isoprenaline on lipolysis and gene expression were due to β_1_‐adrenoceptors, that we showed previously compensate for the lack of β_3_‐adrenoceptors even in brown adipocytes derived from β_3_‐adrenoceptor knockout mice.^23^ However, care should be used when interpreting data derived from human brown adipocyte cell cultures,^33^ as we have previously shown^34^ that in general they express much lower levels of β_3_‐adrenoceptor mRNA than native BAT. This may pose challenges when using immortalized human brown adipocytes to investigate the role of β_3_‐adrenoceptors in human physiology.

Overall, mirabegron is a β_3_‐selective adrenoceptor agonist at the human receptor, as previously shown using cloned receptors.^4^ This contrasts with other agonists previously trialed for treatment of obesity and/or diabetes. One early study using BRL35135, an esterified prodrug that is quickly converted to BRL37344 in vivo, showed that chronic (10 days) administration of BRL35135 in obese subjects improves glycemic control, reduces insulin resistance (improved glucose tolerance profiles), and increases insulin‐mediated body glucose disposal under hyperinsulinemic‐euglycemic clamps.^35^ However, there is convincing evidence that these effects of BRL35135 and its active metabolite BRL37344 primarily result from direct actions at β_2_‐adrenoceptors in skeletal muscle.^36^, ^37^ To examine whether mirabegron acts in vivo on β_1/2_‐adrenoceptors, we performed glucose tolerance tests in β_1/2_‐adrenoceptor knockout mice. Mirabegron still lowered blood glucose levels in these mice, findings that together with the increases in glucose uptake in skeletal mice in wild‐type mice suggest that the improved glucose‐clearing actions of mirabegron in mice are due to activation of β_3_‐adrenoceptors.

There is tremendous interest in the role of brite adipocytes, defined as brown‐like adipocytes located predominately in WAT depots. These adipocytes differ from brown adipocytes in that they originate from a white adipocyte precursor cell, and are converted to brite adipocytes in vitro and in vivo by environmental stimuli such as cold exposure, or a range of pharmaceutical agents.^8^ Brite adipocytes have been advocated as a target for the treatment of metabolic disease, including obesity and type 2 diabetes, and are linked to obesity resistance in several mouse models.^38^, ^39^, ^40^ Here we used rosiglitazone, a PPARγ agonist, to convert white adipocytes to brite adipocytes in vitro, as shown previously.^10^, ^26^ There were minimal effects of mirabegron to increase OCR levels or glucose uptake in white adipocytes, consistent with the low expression of β_3_‐adrenoceptors in these cells and their low levels of UCP1 and mitochondrial content.^10^ However, in brite adipocytes, mirabegron increased cyclic AMP levels, UCP1 mRNA levels, UCP1‐mediated oxygen consumption, glucose uptake, and glycolysis in vitro, consistent with our previous study using β_3_‐adrenoceptor agonists, such as CL316243, or noradrenaline.^10^ It has been proposed that human BAT is actually comprised of brite adipocytes,^41^ and further studies have shown that depending on the location in the body, humans most likely possess both BAT and brite adipocytes.^42^, ^43^ One recent study ^12^ showed that mirabegron (2 mg/kg by osmotic mini‐pumps) resulted in a reduction in body weight, fat mass, and improved glucose tolerance tests in mice fed a high‐fat diet that was associated with the browning of inguinal WAT. In obese humans, administration of mirabegron at 50 mg per day for 12 weeks has been reported to increase BAT volume resulting in improvements in oral glucose tolerance and insulin resistance without weight loss.^15^ In the same study, an increased browning of subcutaneous white fat was reported that was associated with improved insulin sensitivity suggesting that these effects could be due to brite adipose tissue expansion and the induction of both UCP1‐dependent and ‐independent mechanisms. We have shown that mirabegron treatment results in significantly improved glucose clearance primarily through the activation of β_3_‐adrenoceptors. The presence of UCP1 is essential for this effect at least in lean mice housed at sub‐thermoneutral temperatures. It has been recently reported that UCP1 knockout mice exhibit lower glucose uptake in thermogenic tissues with compensatory increases in WAT and skeletal muscle in an oral glucose tolerance test.^44^ Our results imply that these compensatory mechanisms, if further induced by mirabegron in UCP1 knockout mice, are unable to significantly affect postprandial glucose uptake in these tissues to result in substantial increases in glucose tolerance. Although UCP1 is absent in BAT in UCP1 knockout mice, and the acute effects of mirabegron on blood glucose are attenuated, chronic administration may still lead to some induction of UCP1 and possibly other UCP1‐independent mechanisms in brite adipocytes. This notion is supported by the observation that chronic mirabegron treatment of obese subjects, who have very little BAT, still led to improvements in glucose tolerance and insulin resistance,^15^ although no effects on weight loss were shown. The lack of effect on weight loss was speculated^15^ to be due to the relatively low levels of BAT in humans; the dose of mirabegron used in the study (50 mg/day); that this dose of mirabegron was insufficient for browning of WAT and activation of UCP1; or that humans compensate for increased energy expenditure by increased food intake.

There is also great interest in pharmacological/environmental stimuli that can convert white adipocytes to brite adipocytes, as a means to combat obesity. One limitation is that while a range of agents ^8^ can increase the appearance of brite adipocytes, these adipocytes do not become thermogenic until activated by β_3_‐adrenoceptor activity. To increase UCP1 mRNA/protein levels, we treated white adipocytes with rosiglitazone (a PPARγ agonist) to produce brite adipocytes that could then be activated by β_3_‐adrenoceptor ligands.^10^, ^26^ Advantages of pursuing mirabegron for the treatment of obesity/diabetes include that it is well tolerated with a high level of adherence, is orally bioavailable, and has already met requirements set out by regulatory bodies for use in humans. Furthermore, there is a growing body of literature that suggests mirabegron administration results in a pronounced improvement in various homeostatic parameters associated with metabolic syndrome.^15^, ^45^

### 4.1 Perspectives and Significance

In conclusion, our study shows that mirabegron is a β_3_‐selective adrenoceptor agonist and has direct β_3_‐adrenoceptor‐mediated effects on adipocytes that include increases in cAMP, glucose uptake, ECAR, UCP1 mRNA, and OCR. This results in increased adipose tissue glucose uptake, whole body oxygen consumption, and improved glucose tolerance. Additionally, UCP1 is essential for the acute effect of mirabegron on glucose tolerance in vivo.

Most previous studies investigating β_3_‐adrenoceptors in mouse adipose tissue/adipocytes have utilized the β‐adrenoceptor agonist CL316243. While this is a potent and selective β_3_‐adrenoceptor agonist in rodents, it has limited actions at even the cloned human β_3_‐adrenoceptor, and has poor bioavailability, preventing its use in humans. Conversely, while mirabegron is a safe orally active β_3_‐adrenoceptor agonist that is used clinically for overactive bladder, there is limited publically available information on its metabolic actions. This study demonstrates that mirabegron displays very similar properties to CL316243 with respect to cAMP, glucose uptake, ECAR, UCP1 mRNA, and OCR in brown, white, and brite adipocytes, and increased adipose tissue glucose uptake, whole body oxygen consumption, and improved glucose tolerance in vivo. Therefore, mirabegron displays many of the positive metabolic effects of CL316243, is a β_3_‐selective adrenoceptor agonist, and is known to be active in humans suggesting that chronic administration may be useful in the treatment of obesity and diabetes.

## DATA SHARING AND DATA ACCESSIBILITY

The data that support the findings of this study are available from the corresponding author upon reasonable request.

## CONFLICT OF INTEREST

TB owns stocks in the following pharmaceutical companies: Sigrid Therapeutics AB, Atrogi AB, and Glucox Biotechnology AB. DSH owns stocks in Glucox Biotechnology AB and is a consultant for Atrogi AB. RJS owns stocks in Atrogi AB and is a consultant for Servier Laboratories and Atrogi AB.

## AUTHOR CONTRIBUTIONS

DSH performed the majority of the experiments with assistance from ND, MS, MHB, AK, SH, JG, MHN, LW, SM, JM, and LYC. DSH, MHB, RJS, BAE, and TB contributed to the interpretation of the results. DSH and TB designed the study. DSH wrote the manuscript with input from MHB, DW, RJS, BAE, and TB. All authors discussed the results and commented on the manuscript.
